# Correlation of Interleukin-33/ST2 Receptor and Liver Fibrosis Progression in Biliary Atresia Patients

**DOI:** 10.3389/fped.2019.00403

**Published:** 2019-10-01

**Authors:** Jia Liu, YiFan Yang, Chao Zheng, Gong Chen, Zhen Shen, Shan Zheng, Rui Dong

**Affiliations:** Shanghai Key Laboratory of Birth Defect, Department of Pediatric Surgery, Children's Hospital of Fudan University, Shanghai, China

**Keywords:** biliary atresia, interleukin-33, ST2 receptor, liver fibrosis, mast cell, prognosis

## Abstract

**Background/Aims:** Biliary Atresia (BA) is a devastating pediatric liver disease and characterized by aggressive liver fibrosis progression. The Interleukin-33 (IL-33)/ST2 receptor signaling axis has been demonstrated to be involved in several autoimmune and liver diseases. Since immune dysregulation is a contributor to BA pathogenesis, we aimed to investigate the role of IL-33/ST2 receptor in the progression of liver fibrosis in BA patients.

**Materials and Methods:** The study included 36 BA patients (18 good- and 18 poor-prognosis BA patients); and 8 cholestasis infants as the control group. Patients' information and clinical data were retrospectively collected and compared. Liver fibrosis stage was determined by Masson's trichrome staining. Gene expression levels of IL-33, ST2 receptor, and TFG-β1 were detected by quantitative real-time PCR. MC count, IL-33, TGF-β1, and Interleukin-13 (IL-13) expressions were evaluated by immunohistochemistry. Serum IL-33 expression level was detected by enzyme-linked immunosorbent assay. Co-expression of MC and ST2 receptor was detected by immunofluorescence. *In vitro* mast cell was cultured with IL-33 stimulation, and ST2 receptor and TGF-β1 expressions were detected.

**Results:** Compared with cholestasis control, BA patients had significantly higher GGT level and Masson score. Expression levels of IL-33, TGF-β1, and IL-13 were significantly increased in BA patients compared to control group, especially in poor-prognosis BA patients. Co-expression of ST2 receptor and MC was found in BA liver tissues. The MC count was markedly higher in BA patients especially in poor-prognosis subgroup. Serum IL-33 level was significantly elevated in poor-prognosis BA patients and related to a higher Masson score. *In vitro* mast cell culture exhibited significant upregulation of ST2 receptor and TGF-β1 mRNA expression after IL-33 stimulation.

**Conclusions:** IL-33/ST2 receptor signaling axis is correlated with liver fibrosis progression in BA patients, and mast cells participates in this process. These indicate potential prognostic evaluation factors for BA patients and can help in the postoperative management to achieve better long-term prognosis in BA patients.

## Introduction

Biliary atresia (BA) is a leading cause of neonatal obstructive jaundice, characterized by progressive intrahepatic and extrahepatic biliary system obliteration and progressive liver fibrosis. Globally, BA is most common in East Asia, with a frequency of 1.7–3.7 in 10,000 live birth (1). Without prompt and proper treatment, few patients survive past 2-years of age (2). Kasai hepatoportoenterostomy can remove the bile duct remnant, establish bile flow, and has been regarded as the standard surgical treatment for BA. However, many postoperative patients suffer liver fibrosis progression and require liver transplantation (3). The etiology of BA is still unclear, while many hypotheses have been established over the past decades, including the investigation of immunologic abnormalities (3). Since the liver fibrosis progression in BA is more rapid and aggressive than in other chronic liver diseases, the role of inflammatory and fibrotic cytokines has drawn much attention (4).

Interleukin-33 (IL-33), a member of the IL-1 superfamily, is released by injured or necrotic cells and plays an important role in inflammation and immunoregulation (5–8). IL-33 binds specifically to ST2 receptor and induces the production of cytokines and chemokines in various immune cells (5, 8–11). One of the most studied immune cells that express ST2 receptor is mast cell (MC) (10, 12). Previous studies have identified MC in multiple organs including liver (13–17). After being activated by IL-33, hepatic MC releases numerous inflammatory mediators, among which transforming growth factor-beta 1 (TGF-β1) and IL-13 are correlated with liver fibrosis (12, 14, 18, 19).

In BA patients, IL-33 and ST2 receptor were found to be elevated compared with healthy controls (7). Moreover, hepatic MC in fibrotic tissue was demonstrated to be increased with disease progression (17). Therefore, we aimed to investigate the function of the IL-33/ST2 receptor signaling axis in BA patients compared to cholestasis infants with similar symptoms; and to elucidate its correlation with liver fibrosis progression in BA patients of different prognosis. Furthermore, we explored the role of mast cells during this process.

## Materials and Methods

### Patients' Information

This study was approved by the Ethics Committee at the Children's Hospital of Fudan University. We retrospectively included 36 BA patients and 8 cholestasis infants admitted to the department of pediatric surgery in Children's Hospital of Fudan University during June 2016 to March 2019. The diagnoses of BA or cholestasis were based on clinical, cholangiogram, and histological findings. The eight cholestasis infants were included as the control group—they were jaundice patients suspected of BA but excluded from BA diagnosis. The 36 BA infants consisted of 18 good-prognosis (g-BA) and 18 poor-prognosis (p-BA) type-III BA patients who underwent Kasai hepatoportoenterostomy. Grouping was defined by the outcomes at the end of the first year after surgery, the good-prognosis was defined as native liver survival without jaundice (total bilirubin <20 μmol/L), while the poor-prognosis included persistent jaundice, liver transplantation, and death.

All patients' information and clinical data were retrospectively collected, and written consent was obtained from all patients' legal guardians before enrollment. Liver function tests were taken 1–2 days before surgery and at the scheduled follow-ups, including: total bilirubin (TB), direct bilirubin (DB), aspartate transaminase (AST), alanine transaminase (ALT), gammaglutamyl transpeptidase (GGT), total bile acid (TBA), and albumin (Alb).

### Histological Analysis

Liver tissue samples were collected at surgery, fixed in 10% neutral-buffered formalin, embedded in paraffin, sectioned by 4 μm, and stained with Masson's trichrome by standard procedures, or prepared for further experiments. Three experienced pathologists blinded to all specimens evaluated the staging of liver fibrosis using a scoring system based on previous studies (20).

### Quantitative Real-Time Polymerase Chain Reaction (qPCR)

Total RNA was extracted using E.Z.N.A. Total RNA Kit I (Omega Bio-tek, Norcross, USA) and quantified by NanoVue spectrophotometer (Thermo Scientific, Waltham, MA) according to the manufacturer's protocols. cDNA was synthesized using PrimeScript™ RT reagent Kit (TaKaRa, Japan). Real-time PCR was performed by Roche (Thermo Scientific, Waltham, MA) using SYBR Green (TaKaRa, Japan) medium system. *IL-33 and ST2* gene expressions were measured, and *IL-13* and *TFG*-β*1* gene expressions were measured as downstream inflammatory and fibrotic markers. The expression of each gene was normalized to the housekeeping gene *Glyceraldehyde-3-Phosphate Dehydrogenase (GAPDH)*. Primer sequences were verified in nucleotide BLAST database (Table 1). Results were analyzed and normalized to the control group. Experiments were performed in technical and biological triplicates.

**Table 1:** Primer genes used in running quantitative reverse transcription polychain reaction (qRT-PCR) along with their forward and reverse sequences.

**Primer**	**Forward**	**Reverse**
*GAPDH*	5′-GGGGAAGGTGAAGGTCGGAG-3′	5′-CCTGGAAGATGGTGATGGGA-3′
*IL-13*	5′-GAGGATGCTGAGCGGATTCTG-3′	5′-CACCTCGATTTTGGTGTCTCG-3′
*IL-33*	5′-TTGGCATGCAACCAGAAGTC-3′	5′-CCTGTCAACAGCAGTCTACT-3′
*ST2*	5′-ATGGGGTTTTGGATCTTAGCAAT-3′	5′-CACGGTGTAACTAGGTTTTCCTT-3′
*TGF-β1*	5′-CAATTCCTGGCGATACCTCAG-3′	5′-GCACAACTCCGGTGACATCAA-3′

### Immunohistochemistry (IHC)

IHC was performed by a two-step EnVision/HRP technique (Dako Cytomation, Denmark) according to the manufacturer's instructions. The following antibodies (Abcam, Cambridge, UK) were used for IHC: anti-human IL-33 monoclonal antibody diluted to 1:200; anti-human mast cell monoclonal tryptase diluted to 1:400; anti-human TGF-β1 polyclonal antibody diluted to 1:200; and anti-human IL-13 polyclonal antibody diluted to 1:200. All dilutions were made in 3% bovine serum albumin (BSA). Blank negative controls were conducted using 3% BSA instead of antibodies. Cell nuclei were counterstained using hematoxylin. For staining assessment, cells with brown-stained cytoplasm were defined as positive. A digital camera (Leica DM microscope) was used to take images of three randomly selected microscopic fields (leftup, middle, and rightdown, 20 × 10) for each slide. Quantitation of IL-33, TGF-β1, and IL-13 tissue expression was assessed by the size of positive staining area and relative to the control group, using Image-Pro Plus 6.0 software (Media Cybernetics, Bethesda, MD, USA). The expression of MC in BA liver tissue was assessed by MC count in the portal area.

### Immunofluorescence (IF)

IF was applied for detecting MC expression and its co-expression with ST2 receptor. A monoclonal mouse anti-human mast cell tryptase (Abcam, Cambridge, UK) diluted to 1:400 and a polyclonal goat anti-human ST2 antibody (Abcam, Cambridge, UK) diluted to 1:100 in 5% BSA were used, followed by eflour 555–conjugated donkey anti-mouse and eflour 488-conjugated donkey anti-goat antibodies diluted to 1:500 (Thermo Fisher Scientific, Hudson, NH, USA). Slides were examined by Leica DM microscope (20 × 10).

### Enzyme-Linked Immunosorbent Assay (ELISA)

Human serum samples were obtained from blood samples during surgery, by centrifuging at 3,000 rpm for 10 min and stored at −80°C for future studies. ELISA kit (RayBiotech, Norcross, USA) was used to detect the IL-33 expression levels in patients' serum according to the manufacturer's protocol. Based on the circumstances during surgery and sample processing, the human serum samples were not complete for all the included patients, while the sample size was 13 in the g-BA group and 14 in the p-BA group. The standard curve and all samples were processed in duplicate and the experiment was repeated once.

### Human Mast Cell Culture

Human mast cells HMCs-1 were obtained from the Institute of Biochemistry and Cell Biology at the Chinese Academy of Science (TCHu69, Shanghai, China) and cultured in DMEM medium supplemented with 10% FBS, 100 IU/ml penicillin, and 100 mg/ml streptomycin sulfates at 37°C with 5% CO_2_. HMCs-1 were seeded into 24-well culture plates at 2 × 10^5^ cells/well; 0.4 μg/mL recombinant human IL-33 (Peprotech, Princeton, USA) was added at the same time, while an equal amount of DMEM medium was added as blank control. After 24 h of stimulation, cells were harvested and processed for further experiments. Experiments were assayed in technical and biological triplicate.

### Statistical Analysis

Data were expressed as mean ± standard deviation (SD). Student's *t*-test and Chi-Square (or Fisher's exact) test were used for comparisons among groups as appropriate. All analyses were performed using GraphPad Prism 6 software and SPSS 21.0 software. A *p* < 0.05 was considered statistically significant.

## Results

### Patients' Information

The patients' information and clinical data of control and BA groups are shown in Table 2. Compared with the control group, BA patients have significant higher GGT level (719.9 ± 91.6 vs. 160.8 ± 46.6 IU/L, *p* = 0.0070), lower Alb level (38.5 ± 0.6 vs. 41.3 ± 1.3 g/L, *p* = 0.0425) before surgery, and higher Masson score (2.1 ± 0.1 vs. 1.1 ± 0.1, *p* = 0.0002) at surgery. This indicates a higher level of liver fibrosis and dysfunction in BA patients.

**Table 2:** Patients' information of control and BA groups.

	**Control**	**BA**	***P*-value**
Age (day)	75.4 ± 11.4	63.3 ± 3.3	0.1731
Male/Female	7/1	22/14	0.2308
TB (μmol/L)	173.1 ± 30.9	164.2 ± 6.8	0.6612
DB (μmol/L)	119.9 ± 19.6	105.4 ± 4.2	0.2546
AST (IU/L)	228.7 ± 50.4	175.8 ± 18.9	0.2617
ALT (IU/L)	134.5 ± 29.8	108.1 ± 10.6	0.3238
GGT (IU/L)	160.8 ± 46.6	719.9 ± 91.6	0.0070*
TBA (μmol/L)	133.1 ± 36.0	150.3 ± 8.9	0.4966
Alb (g/L)	41.3 ± 1.3	38.5 ± 0.6	0.0425*
Masson score	1.1 ± 0.1	2.1 ± 0.1	0.0002*

* *p < 0.05*.

Among BA patients, the jaundice clearance time in g-BA group on average is 2.7 ± 1.4 months, while no patient achieved jaundice clearance in the p-BA group. Among p-BA patients, nine died at an average of 6.6 ± 2.3 months, two received liver transplantation at 6–7 months after Kasai procedure, and seven exhibited persistent jaundice and awaited liver transplantation at 1-year after Kasai procedure. There is no significant difference in patients' information or clinical data between the g-BA and p-BA group (Table 3).

**Table 3:** Patients' information of g-BA and p-BA groups.

	**g-BA**	**p-BA**	***P*-value**
Age (day)	61.9 ± 4.6	64.6 ± 4.8	0.6991
Male/Female	10/8	12/6	0.7332
TB (μmol/L)	168.0 ± 11.3	160.5 ± 7.7	0.5853
DB (μmol/L)	106.1 ± 6.7	104.6 ± 5.1	0.8649
AST (IU/L)	176.6 ± 25.9	175.0 ± 28.4	0.9679
ALT (IU/L)	106.7 ± 15.8	109.5 ± 14.7	0.8962
GGT (IU/L)	707.9 ± 115.8	731.8 ± 145.4	0.8985
TBA (μmol/L)	163.6 ± 11.6	137.0 ± 13.0	0.1358
Alb (g/L)	39.3 ± 0.9	37.6 ± 0.7	0.1230
Masson score	2.1 ± 0.2	2.1 ± 0.2	0.8016

### IL-33/ST2 Receptor Elevation in BA Patients

**Figure 1:**
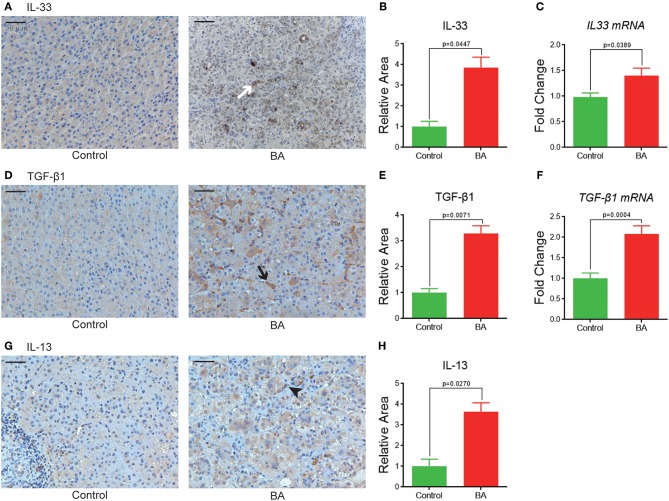
IL-33, TGF-β1, and IL-13 expressions in BA livers. **(A)** IL-33 expression in control and BA livers, shown as brown positive staining (white arrow). Quantification of IL-33 expression by IHC **(B)**, and qPCR **(C)**. **(D)** TGF-β1 expression in control and BA livers, positive staining shown as black arrow. Quantification of TGF-β1 by IHC **(E)** and qPCR **(F)**. **(G,H)** IL-13 IHC and quantitation in control and BA livers, positive staining shown as black arrowhead.

As a first step in determining the relevance of a IL-33/ST2 signaling axis in BA, IHC, and qPCR was performed for targets implicated in this pathway. IHC analyses showed that compared with the control group, BA patients have significant higher expression levels of IL-33 (3.9 ± 0.5 vs. 1.0 ± 0.3, *p* = 0.0447, Figures 1A,B), TGF-β1 (3.3 ± 0.3 vs. 1.0 ± 0.2, *p* = 0.0071, Figures 1D,E), and IL-13 (3.6 ± 0.4 vs. 1.0 ± 0.3, *p* = 0.0270, Figures 1G,H). Similarly, gene expression levels of *IL-33* (1.4 ± 0.1 vs. 1.0 ± 0.1, *p* = 0.0389, Figure 1C), *ST2 recepto*r (1.6 ± 0.2 vs. 1.0 ± 0.2, *p* = 0.0336, Figure 3D), and *TGF*-β*1* (2.1 ± 0.2 vs. 1.0 ± 0.1, *p* = 0.0004, Figure 1F) are significantly elevated in BA livers compared to control livers, while *IL-13* mRNA expression is undetectable in our experiments.

**Figure 2:**
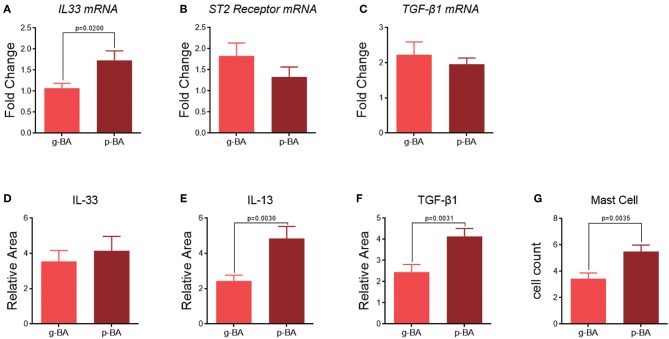
Subgroup analyses between g-BA and p-BA groups. mRNA expression levels of *IL-33* **(A)**, *ST2 receptor* **(B)**, and *TGF*-β*1* **(C)** in g-BA and p-BA livers. Quantification in IHC staining of IL-33 **(D)**, IL-13 **(E)**, and TGF-β1 **(F)** expression in g-BA and p-BA livers. **(G)** Mast cell count in the liver of two BA subgroups.

In the subgroups of BA patients, IHC staining shows IL-33 expression level is relatively higher in p-BA livers compared to g-BA livers (4.1 ± 0.8 vs. 3.6 ± 0.6, *p* > 0.05, Figure 2D), while its mRNA expression level is statistically significant (1.7 ± 0.2 vs. 1.1 ± 0.1, *p* = 0.0200, Figure 2A). The downstream cytokines TGF-β1 and IL-13 expression levels were also significantly elevated in p-BA livers compared to g-BA livers in IHC staining (TGF-β1 4.1 ± 0.4 vs. 2.5 ± 0.4, *p* = 0.0031; IL-13 4.8 ± 0.7 vs. 2.4 ± 0.3, *p* = 0.0030, Figures 2E,F). While the difference in the gene expression of *ST2 receptor* or *TGF*-β*1* is not significant between the two BA subgroups (Figures 2B,C).

### IL-33 Is Related to BA Prognosis and Liver Fibrosis

**Figure 3:**
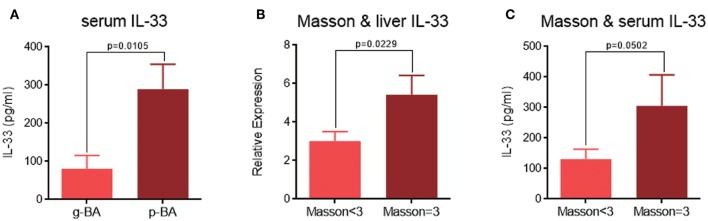
The relation of IL-33 expression levels with BA prognosis and liver fibrosis. **(A)** Serum IL-33 expression levels between g-BA and p-BA groups. **(B)** Liver IL-33 expression levels by IHC in patients with different Masson scores. **(C)** Serum IL-33 expression levels by ELISA in patients with different Masson scores.

When comparing between g-BA and p-BA subgroups, ELISA showed significantly elevated IL-33 levels in the serum of p-BA patients, indicating the predictive property of IL-33 for disease prognosis (289.7 ± 65.0 vs. 80.8 ± 35.0 pg/ml, *p* = 0.0105, Figure 3A). Moreover, when comparing the expression levels of IL-33 among different Masson scores, significant differences were detected between patients with a Masson score of 3 and those with a Masson score of 1 and 2. The IL-33 expression levels in the liver biopsy IHC (relative expression 5.4 ± 1.0 vs. 3.0 ± 0.5, *p* = 0.0229, Figure 3B) and serum (305.1 ± 102.2 vs. 131.1 ± 32.3 pg/ml, *p* = 0.0502, Figure 3C) were significantly higher in patients with a Masson score of 3, indicating that IL-33 can be related to liver fibrosis stage.

### Mast Cell Detection and *in vitro* Study

**Figure 4:**
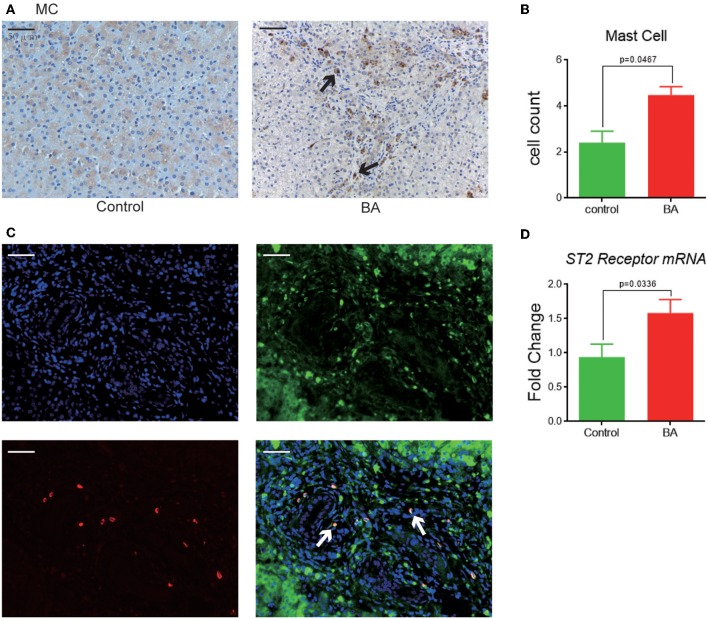
MC expressions and its co-expression with ST2 receptor. **(A)** MC tryptase expression in control and BA livers, shown as brown positive staining (black arrow). **(B)** MC quantification in the liver of two groups by cell count. **(C)** Immunofluorescence of MC tryptase (red), ST2 receptor (green), and DAPI (cell nuclei). Merged figure shows co-localization of MC and ST2 receptor (white arrows). **(D)** ST2 receptor mRNA expression in control and BA livers.

MCs are mostly detected in the portal area of BA livers (Figure 4A), with a significant higher cell count compared to the control livers (4.5 ± 0.4 vs. 2.4 ± 0.5, *p* = 0.0467, Figure 4B). When compared between the BA subgroups, MC count is higher in the p-BA livers than that in the g-BA livers (5.5 ± 0.5 vs. 3.4 ± 0.4, *p* = 0.0035, Figure 2G). Co-expressions of MC and ST2 receptor can be detected in the IF staining in BA livers (Figure 4C). Gene expression level of ST2 receptor is significantly higher in BA livers compared to control livers (1.6 ± 0.2 vs. 0.9 ± 0.2, *p* = 0.0336, Figure 4D).

**Figure 5:**
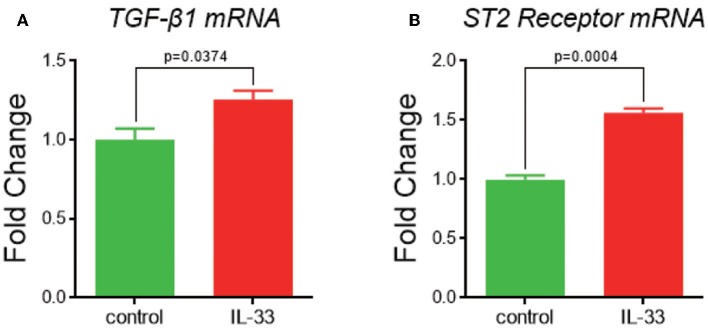
*TGF*-β*1* and *ST2 receptor* expression after IL-33 stimulation in *in vitro* mast cell culture. **(A)** *TGF*-β*1* and **(B)** *ST2* mRNA expression in control and IL-33 stimulated MC.

When given IL-33 stimulation in HMCs-1 for 24 h *in vitro, TGF*-β*1* and *ST2 receptor* mRNA expression levels are significantly increased compared with blank control (*TGF*-β*1* 1.3 ± 0.1 vs. 1.0 ± 0.1, *p* = 0.0374; *ST2* 1.6 ± 0.0 vs. 1.0 ± 0.0, *p* = 0.0004, Figures 5A,B).

## Discussion

**Figure 6:**
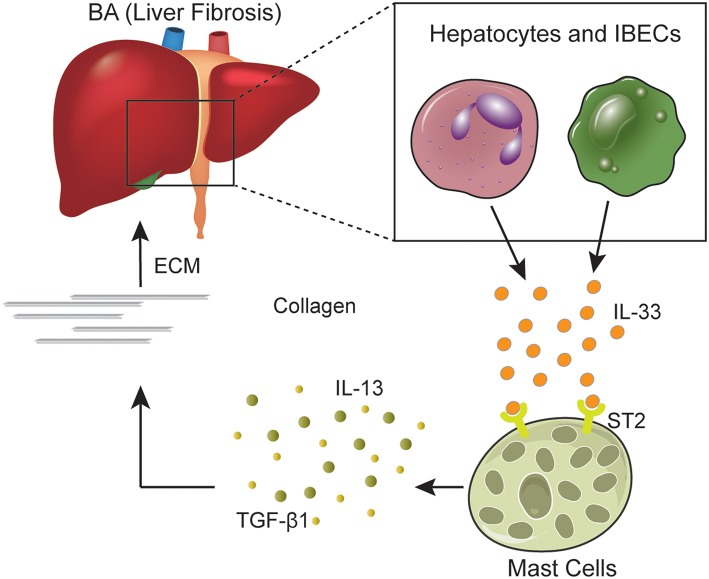
IL-33/ST2 signaling axis. IL-33 is secreted mostly by hepatocytes and intrahepatic biliary epithelial cells in the liver. After combining with ST2 receptor expressed on mast cells and other cells, IL-33 induces mast cells to release inflammation and fibrotic cytokines such as IL-13 and TGF-β1. IL-13 and TGF-β1 mediates the production of collagen and extracellular matrix, which could contribute to liver fibrosis in BA patients. BA, biliary atresia; IBECs, intrahepatic biliary epithelial cells; ECM, extracellular matrix.

In the present study, we found that IL-33/ST2 receptor signaling axis is activated in BA patients, especially in poor-prognosis postoperative BA patients, while mast cells participated in this process (Figure 6). As an “alarm” cytokine, IL-33 has been demonstrated to exacerbate inflammatory responses and drive liver fibrosis (7, 8). Previous studies have shown that IL-33 is associated with tissue damage (21), and IL-33 expression was increased in both BA patients and experimental BA models (7, 22). However, the control groups in previous studies are always healthy controls with normal liver function. In our study, we chose cholestasis infants as the control group. The cholestasis infants in our study had similar symptoms, including abnormal liver function, liver injury, and inflammation, while the major difference from BA patients is less progressive or no liver fibrosis. Therefore, the differences we detected between the two groups could indicate the effects of these factors in the process of liver fibrosis. We also explored the difference between subgroups of BA patients with different prognosis, which made it more convincing to elucidate the role of IL-33/ST2 receptor signaling axis in the liver fibrosis progression.

We found that IL-33 expression is significantly higher in BA patients compared to cholestasis controls and was especially higher in poor prognosis BA patients, indicating that IL-33 is correlated with liver fibrosis progression and can be an indicator for postoperative prognosis. Moreover, we analyzed the relation between IL-33 expression and Masson score, which presenting the liver fibrosis stage. We found out that IL-33 expressions in liver and serum are significantly increased in the poor-prognosis BA patients compared to those of the good-prognosis BA group, indicating that IL-33 can be a possible predictor for liver fibrosis stage.

For the investigation of the expression of downstream cytokines related to the IL-33/ST2 receptor signaling axis, IL-13 is mainly secreted after IL-33 stimulation and can induce epithelial injury and biliary inflammation (3). TGF-β1 is one of the most important liver fibrosis-inducing cytokines and can induce collagen deposition and fibrosis, regulate extracellular matrix formation, degradation, and remodeling (23). TGF-β1 expression has been found to be increased in the liver of primary biliary cirrhosis (PBC), primary sclerosing cholangitis (PSC), BA, chronic viral hepatitis, and alcoholic liver disease, among which is mainly correlated with hepatic fibrosis progression (19, 24). Our study revealed that BA livers had significant higher expression levels of TGF-β1 and IL-13 compared to the cholestasis control. Similarly, the levels of TGF-β1 and IL-13 protein expression are also elevated in poor-prognosis BA patients compared to the good-prognosis BA group. These indicate that TGF-β1 and IL-13 play an important role in both liver inflammation and fibrosis, especially fibrosis progression in BA patients, and higher levels of TGF-β1 and IL-13 before Kasai procedure are correlated with poor prognosis post surgery.

To explore the target for IL-33/ST2 receptor signaling axis, we experimented on mast cells. *In vivo*, similar to previous studies (13), we identified that MCs are mostly located in portal areas, which is also mostly fibrotic tissues, of BA livers. IF staining showed co-localization of ST2 and tryptase, indicating ST2 receptor expressed on MC. However, there are several separate ST2 staining in our study, which can be explained by that ST2 can also be expressed on the surface of some other cell types (10). Though contributing to BA progression to some extent, MC is demonstrated to play an important role in autoimmune diseases and is increased in other chronic liver diseases such as PBC and PSC (25–27). During liver fibrosis, MC can enhance extracellular matrix production, recruit matrix-producing cells and stimulate cytokine secretion (13, 14, 28). In agreement, we found that MC count and ST2 receptor expression were significantly higher in BA livers compared to control livers. Furthermore, we discovered that MC count was significantly higher in p-BA livers compared to g-BA, indicating the role of MC during the progression of liver fibrosis in BA.

To elucidate the interaction between IL-33/ST2 receptor signaling axis and MC in BA, we did *in vitro* experiments on HMCs-1. After 24 h of stimulation by IL-33, cultured MC showed increased expression levels of *ST2 receptor* and downstream cytokine *TGF*-β*1*, compared to control. Therefore, we hypothesize that the elevated IL-33 in BA livers associates with ST2 receptor and subsequently stimulates MC function, ultimately contributing to the progression of liver fibrosis.

There are several limitations in our study. First, the sample size of control group was relatively small, mainly due to the difficulty in collecting liver tissue samples in this group. Moreover, ST2 receptor is also expressed on other immune cells, which should be investigated in future studies.

In conclusion, our study showed that compared to cholestasis controls, IL-33 and ST2 receptor expressions are significantly increased in BA livers; their interaction can induce the overexpression of downstream cytokines TGF-β1 and IL-13. In BA patients, IL-33, TGF-β1, and IL-13 expressions are correlated with postoperative prognosis. This indicates an important role of the IL-33/ST2 receptor signaling axis in the progression of liver fibrosis in BA patients, and provides evidence for the capability of IL-33 being a prognostic biomarker. Furthermore, we found out that mast cells partially participated in this process. These findings can provide prognostic evaluation factors for postoperative BA patients, and will help to achieve an earlier detection of those patients who have more progressive liver fibrosis and are at higher risk of liver transplantation. The study hereby is of importance in the postoperative individualized management and the achievement of better long-term prognosis in BA patients.

## Data Availability Statement

The datasets generated for this study are available on request to the corresponding author.

## Author Contributions

JL, RD, and SZ: study conception and design. JL, YFY, CZ, GC, and ZS: data acquisition. JL, YFY, CZ, and RD: analysis and data interpretation and drafting of the manuscript. GC, ZS, and SZ: critical revision.

### Conflict of Interest

The authors declare that the research was conducted in the absence of any commercial or financial relationships that could be construed as a potential conflict of interest.
